# Antimicrobial activity of tea tree and lavender essential oils and their effects on hatching performance and eggshell bacterial count of Japanese quail eggs

**DOI:** 10.1186/s12917-025-04576-4

**Published:** 2025-03-17

**Authors:** Ebtsam E. Iraqi, Amany A. EL-Sahn, Amal M. EL-Barbary, Mona M. Ahmed, Alaa E. Elkomy

**Affiliations:** 1 Poultry Breeding Research Department, Animal Production Research Institute, Agriculture Research Center, Ministry of Agriculture, Dokii, Giza, 12611 Egypt; 2 Livestock Research Department, Arid Lands Cultivation Research Institute, City of Scientific Research and Technological Applications (SRTA-City), New Borg El Arab, 21934 Egypt; 3 Faculty of Desert and Environmental Agriculture, Matrouh University, Matrouh, 51512 Egypt

**Keywords:** Lavender oil, Tea tree oil, Fertilized egg, Formaldehyde fumigation and hatchability

## Abstract

The objective of this work was to study the effect of tea tree (TTO) and lavender (LavO) essential oils instead of formaldehyde fumigation to disinfect the surface of fertilized eggshells. A total of 1050 fresh unwashed fertilized quail eggs were randomly divided into 7 groups and treated before incubation as follows: group 1 was untreated (negative control), group 2 was sprayed with 70% ethyl alcohol (positive control), group 3 was fumigated with formaldehyde gas (FF), groups 4–5 and 6–7 were sprayed with 2%, 3% TTO and 2%, 3% LavO, respectively. Spraying fertilized eggs with 3% TTO or LavO significant reduced (*P* < 0.05) egg weight loss and improved yolk sac absorption expressed as a decrease in the yolk sac remaining weight (*P* < 0.01), which coincided with increasing the percentage of embryonic weight at 14th day of incubation compared to the FF. TTO or LavO significantly boosted the hatching rate that correlated with significant reduction (*P* < 0.01) in embryonic mortality with preference for LavO. Spraying fertilized eggs immediately after collection with TTO or LavO significantly reduced (*P* < 0.01) total bacterial count on the eggshell surface compared to the FF. Thus, TTO and LavO can be used to disinfect fertilized eggs prior to incubation to improve hatching rates and chicks’ quality upon hatching.

## Introduction

The first and most important stage in poultry industry is hatchability and improving hatchability characteristics is a very critical step to improve poultry production efficiency [1]. Maximizing the efficiency of egg incubation operations and improving the quality of day-old chicks are the main goals of broiler farming [2].

Eggs exposed to multiple microbial contaminants during egg laying such as E. coli, salmonella, enteric bacteria, yeasts and molds, which can penetrate the eggshell [3]. Contamination of fertilized eggs with microorganisms is becoming more important, which may cause injury to the fetus, leading to decreased hatching efficiency. This effect is due to the fact that the ideal environment for fetal development is the same as that required for microorganism multiplication [4]. Thus, it is necessary to have an effective hatchery sanitation program before starting egg incubation to prevent growth and reduce the content of pathogenic microorganisms on the surface of eggshells, which are extremely harmful to embryonic development, leading to improved hatching efficiency [4, 5]. To achieve this goal, fumigation of hatching eggs using paraformaldehyde is commercially used by most producers [6]. In spite of, this technique is efficient to reduce the harmful hazardous of the potential pathogenic microorganisms [7] but formaldehyde is toxic and has adverse effects not only for chick embryos but also for human health and environment [8, 6].

Hence, alternative products are needed to provide safe and satisfactory sanitation for incubation efficiency and human health. Natural products that derived from plants are possess various therapeutic properties. The natural products derived from plants are composed of many biologically active compounds, which have biological activity against several disease-causing agents [9] and are generally thought to be more acceptable and less hazardous than synthetic compounds [10].

Usually, liquid essential oils are complex combinations of bioactive ingredients of lipophilic substances derived from secondary metabolites of plants. Chemically, most essential oils consist of terpenoids, phenylpropanoids, alkenes and linear alkanes [11]. The efficient antimicrobial properties of essential oils may help solve the problems of high bacterial resistance and the increased costs of using new generations of antibiotics [12]. Essential oils represent alternative sources and environmental control agents for infectious organisms due to their antimicrobial properties [13]. Interestingly, Elkomy et al*.,* [14] showed that essential oils such as grape seed and aniseed significantly reduce pathogenic microbes (such as *E. coli* and *Salmonella* spp.) and promote the growth of beneficial bacteria (such as *Lactobacillus* spp.), therefore, it can be replaced instead of antibiotics of growing quail’s diet.

Recently, natural products have been used as alternatives to formaldehyde in many studies, such as clove essential oil, which may be an alternative to sterilizing fertilized eggs [2], etheric thyme oil which has antimicrobial properties [13, 15], propolis (a resin-like material made by bees) [16], allicin [17] and garlic oil [18]. The study of Copur et al*.,* [17] showed that it is safe to treat broiler eggs with allicin (3600 mg/L and 7200 mg/L) as an alternative to formaldehyde fumigation and resulted in improved hatchability, reduced contamination and embryonic mortality rates. Similarly, immersion quail eggs in garlic extract in concentration 2.5 and 5.0% [18] or spraying garlic oil solution in concentration 1 ml/l or 2 ml/l [19] as an alternative to formaldehyde fumigation method before incubation improved embryonic development, hatchability and chick’s hatch weight.

Lavender (*Lavandula Angustifolia*) is a powerfully aromatic shrub, belonging to the *Lamiaceae* family that grows in various regions around the world. Lavender essential oil (LavO) is extracted from lavender flowers, has healing properties such as antimicrobial and antifungal [20] and antioxidant activity, which is particularly attributed to the presence of phenolic and polyphenolic substances [21, 22] and is used as a natural preservative in the food industry [20]. The main active compounds in LavO are linalool, linalyl acetate, alpha-terpineol, lavandolyl acetate, caryophyllene, geranyl acetate and terpinen-4-ol [23, 24] and camphor, 1,8-cineole and β-cymene [25]. According to Carrasco et al*.,* [26] and Adaszyńska-Skwirzyńska and Szczerbińska [24], oxygenated monoterpenes, such as linalool and linalool oxide have antioxidant beside varied antimicrobial properties, so effective antimicrobial and antifungal agents may include LavO.

*Melaleuca alternifolia*, commonly known as the tea tree is a species of tree or tall shrub from the myrtle family (*Myrtaceae*). It grows along streams and in swampy areas and is often the dominant species in these ecosystems. Natural materials extracted from the tea tree are commonly used in the pharmaceuticals production as an alternative medicinal plant in Australia [27]. According to the study of Elmi et al*.,* [28] the main chemical composition of TTO was terpinen-4-ol, γ-terpinene, α-terpinene and α-terpineol, beside these four components it contains 1,8-cineole, p-cymene and α-pinene and the major sensitizers appear to be ascaridole, terpinolene, α-terpinene, α-phellandrene, 1,2,4-trihydroxymenthane and limonene [29]. Due to the increasing incidence of infections resistant to antibiotics or chemotherapy, TTO can be used as an alternative treatment or in combination with conventional drugs to enhance their effect [27, 30]. The tea tree essential oil (TTO) has antimicrobial, antioxidant, and acaricidal properties. Due to these properties, it is one of the most important subjects studied [31]. TTO has a broad spectrum of antimicrobial activity against a wide range of bacteria, viruses, and fungi, including yeasts and dermatophytes. The most important component in this oil due to its proven antimicrobial properties is terpinen-4-ol) [27, 32, 33]. According to Mumu and Hossain [34], the tea tree essential oil demonstrated remarkable antibacterial activity and may be effective in fungal infection treatments [35]. The antibacterial properties of TTO are due to the stimulation of cellular potassium ion leakage and inhibition of respiration in E. coli cells, providing evidence for a lethal action linked to cytoplasmic membrane damage [36]. Tea tree essential oil should not be taken in large quantities due to its toxicity, although no fatal cases have been reported in the medical literature [35].

In poultry industry, for the success of hatching stage, we must consider the importance of maintaining the quality of fertilized eggs from the egg laying stage until they are introduced into the hatchery. To achieve this goal, our hypothesis aims to study the essential oils of LavO and TTEO as a natural antimicrobial agent to inhibit the growth and reduce the pathogenic microorganisms count on the surface of eggshells, as well as, reducing the harmful risks resulting from using formaldehyde fumigation technique on hatchery workers.

## Materials and methods

### Breeder flock

A total of 1050 fresh-clean fertilized quail eggs (unwashed, feces-free) were used to investigate the effect of some organic oil (Tea tree essential oil (TTO) and Lavender essential oil (LavO)) to improve egg hatching percentage by reducing eggshell contamination by microorganisms compared to the traditional Formaldehyde fumigation treatment (FF) during the incubation and hatching period. The experiment was conducted at El-Sabahia Poultry Research Station (Alexandria), Animal Production Research Institute, Agricultural Research center Egypt.

The eggs used in this study were taken from a quail (Coturnix coturnix japonica quails) flock consisting of 900 females + 450 males. The quails were housed in a controlled environment house equipped with wire floor battery cages and the sex ratio was 2 females:1 male per cage at 12 weeks of age. The birds were exposed to a photoperiod of 16 h light:8 h darkness daily and the environmental temperature inside the house was approximately 23 ⁰C. The quails were fed a breeder ration containing 2894.42 kcal ME/kg and %19.90 CP (Table 1). Feed and water were provided ad libitum.

**Table 1:** Composition and calculated analysis of the basal experimental diet ingredients

Ingredients	%
Yellow corn	53.58
Soybean meal (48%)	30.50
Di-calcium phosphate	1.16
Limestone	6.50
Lysin	0.02
Wheat bran	4.50
Sunflower oil	3.00
Vit. and minreal. mix.^a^	0.300
Salt (NaCl)	0.300
Methionine	0.140
**Total**	**100**
**Calculated analyses:**	
Crude protein, %	19.90
ME (Kcal/ kg diet)	2894.42
Ether extract, %	2.48
Crude fiber, %	2.74
Methionine, %	0.45
Methionine + cystine, %	0.74
Lysine, %	1.01
Calcium, %	2.82
Av. Phosphorus	0.38

^a^Each kg of vitamin and minerals mixture contained: Vit. A, 4,000,000 IU; Vit. D3, 500,000 IU; Vit, E, 16.7 g., Vit. K, 0.67 g., Vit. B1, 0.67 g., Vit. B2, 2 g., Vit. B 6, .67 g., Vit. B12, 0.004 g., Nicotinic acid, 16.7 g., Pantothenic acid, 6.67 g., Biotin, 0.07 g., Folic acid, 1.67 g., Choline chloride, 400 g., Zn, 23.3 g., Mn, 10 g., Fe, 25 g., Cu,1.67 g., I, 0.25 g.,Se, 0.033 g. and,Mg, 133.4 g

Fertilized eggs were collected four times a day and quickly transferred to a fresh clean room for treatment with the tested materials (ethyl alcohol (EA), tea tree (TTO) and lavender oils (LavO) upon collection immediately before being stored in a cold room (at 18°C ​​and 75% relative humidity) for four days until introduction into the incubator on the fourth day of storage.

### Preparation of solutions

Tea tree oil and lavender oil were purchased from a private Egyptian company (Imtenan Company at San Stefano, Alexandria, Egypt).

To prepare tea tree and lavender oils solutions we used ethyl alcohol 70% solution (prepared by mixing 70 ml ethanol and 30 ml distiller water) as a carrier. It was dissolved 20 ml of TTO or LavO oils in 980 ml of ethyl alcohol 70% to prepare 2% TTO or LavO solutions. As well. It was dissolved 30 ml of TTO or LavO oils in 970 ml of ethyl alcohol 70% to prepare 3% TTO or LavO solutions.

### Application of solutions

A total of 1050 fertile eggs were randomly divided into 7 groups in completely randomized design (150 eggs of each), eggs of each treatment were put in three incubation trays. The first group was untreated that served as a negative control group (control), the second group was sprayed with EA (70%) and served as a positive control. The third group was fumigated with formaldehyde gas (FF) (119.8 ml formalin + 59.9 g potassium permanganate/2.83 m3 for 20 min) according to the method described by Yildirim et al*.,* [37]. The fourth and fifth groups were sprayed with TTO at level 2% and 3%, respectively. The sixth and seventh groups were sprayed with LavO at level 2% and 3%, respectively. The solutions were sprayed onto the egg, using a hand sprayer, to cover the whole surface. The treated eggs were allowed to dry at 22 °C for 10 min.

### Incubation management

Eggs of each treatment were numbered consecutively and weighed before starting incubation. The incubation period was 14 consecutive days at 37.5 ºC and 60–65% RH. All egg trays were set and distributed randomly at different places inside the incubator to reduce the effect of tray position on embryos growth. At the beginning of the 15th day of incubation, the eggs were transferred to the hatchery at 37.2 °C and 75% RH until the chicks hatched.

### Data collected

#### Egg quality assay

At the end of the egg storage period, 10 eggs from each treatment were randomly chosen immediately before egg incubation to evaluate egg quality. Eggs were weighed to the nearest 0.1 g and egg shape index was measured. Eggshell, albumin, and egg yolk weights as a percentage of egg weight were determined. The shell thickness with membrane (mm) was measured at three sites of eggshell using a micrometer. Yolk color intensity was assessed based on the standard color of the yolk using a Roche yolk color fane with a score range of 1–15 from light yellow to the dark yellow. Also, Haugh unit and yolk index were calculated.

#### Hatchability performance

On 14th day of incubation, all eggs were individually weighed to determine egg weight loss. Also, 10 fertile eggs of each treatment were randomly chosen to determine the embryo and remaining yolk sac weight and shell thickness. After hatching was complete (at day 17.5 of incubation), unhatched eggs were taken and opened to determine the stage of embryonic mortality. Embryonic mortality was classified into three stages (1st to 4th day, 5th to 15th day and 16th day to pull out chicks) and the mortality percentage was expressed as percentage of fertile eggs. Fertility percentage was calculated as the percentage of set eggs. Hatchability percentage was expressed as the percentages of fertile eggs. Hatched chicks were weighed and chick quality was assessed according to Tona et al*.,* [38] (chick length, activity, downs and appearance, navel, remaining membrane, remaining yolk). Also, 42 one-day-old quail chicks (6 chicks/ treatment) were slaughtered to weigh the internal organs (liver, gallbladder, gizzard, heart, yolk sac, intestine).

### Microbiological analyses

#### Preparation of samples

Three eggs per group (21 eggs) were taken for microbiological analysis at end of the storage period immediately before incubation for all treatments, the eggs were grouped mainly in pools of 3. The eggs were soaked in 120 ml sterile buffered peptone water 1% (40 ml for each) in a sterile bag. Subsequently, the eggs were rubbed gently through the bag for a minute. Tenfold serial dilutions were prepared from the prepared buffered peptone water for the bacteriological examination [39].

#### Determination of total aerobic bacterial count

One milliliter from each of the previously prepared serial dilutions was transferred aseptically into each of duplicate sterile Petri dishes. About 10–12 ml of sterile melted and cooled at (45 + 1 °C) standard plate count agar medium was poured into each plate and mixed carefully. After solidification, the inoculated plates including control one (inoculated with sterile distal water) incubated at 32 ± 1 °C for 48 ± 3 h [40].

### Statistical analysis

Data were statistically analyzed according to the [41]. Statistical analysis was performed using one-way ANOVA. Significant differences among treatment groups were subjected to Tukey test. Results were considered significant at *P* ≤ 0.05.

The statistical model used was as follows:

$${\text{X}}_{\text{ij}}=\mu +{\text{T}}_{\text{I}}+{\text{e}}^{\text{ij}}$$

where x_ij_ is the value of the measured variable, µ is the overall mean, T_i_ is the effect of treatment (i = 7 treatments), and e_ij_ is the random error.

## Results

Before introducing the fertilized quail eggs into the incubator and starting the incubation period, the egg quality traits were studied for the treated and untreated eggs to determine the effect of treatments with TTO or LavO on the egg quality traits and the data were posted in Table (2). The data revealed no significant differences between eggs treated and untreated with the tested materials regarding the egg weight, eggshell weight, eggshell thickness and egg shape index, as well, the internal traits like yolk and albumin weight, yolk index, Haugh unit and yolk color.

**Table 2:** The effect of spraying fertile quail eggs with tea tree (TTO) and lavender oils (LavO) before incubation on egg quality traits

Traits	Egg weight (g)	Egg shape index (%)	Shell weight (%)	Yolk weight (%)	Albumen weight (%)	Yolk index (%)	Haugh unit	Shell thickness (mm)	Yolk color
Treatment									
Control	11.92	78.85	13.11	35.33	49.56	40.15	87.18	0.24	3.67
EA70%	11.9	79.41	13.47	37.11	49.42	41.07	87.60	0.25	4.33
FF	11.9	79.19	13.94	38.46	47.60	41.98	87.16	0.24	4.33
TTO 2%	11.89	78.54	14.48	36.58	48.93	40.01	87.41	0.23	4.00
TTO 3%	11.89	79.04	13.77	36.58	49.58	41.45	87.78	0.24	4.00
LavO 2%	11.9	78.29	14.59	36.63	48.78	39.56	87.24	0.24	4.00
LavO 3%	11.87	78.45	14.84	36.80	48.35	41.81	87.58	0.23	3.67
SEM	0.45	1.53	0.72	1.61	1.19	1.19	0.71	0.01	0.27
*P*-value	0.992	0.999	0.765	0.944	0.984	0.664	0.995	0.922	0.67

*SEM* Standard error of means, *P* Probability level

Data of TTO and LavO treatments on embryonic development, remaining yolk sac weight, eggshell weight and eggshell thickness on the 14th day of incubation and egg weight loss from the 0 to 14th day of incubation are shown in Table (3). It could be noted that the percentage of egg weight loss during the incubation period (from 0 to 14th day) was lower in the LavO, TTO and FF groups compared to the control group, and this effect was significant (*P* < 0.05) with the 3% LavO and TTO level only. In addition, the comparison between TTO, LavO and FF in regarding to the percentage of egg weight loss during the incubation period (from 0 to 14th day) explained that both LavO and TTO led to a decrease in the percentage of egg weight loss compared to the FF treatment, but this decreasing was non-significant. It is worth noting that spraying eggs with TTO or LavO at any dose led to a significant increase (*P* < 0.01) in the embryonic weight on the 14th day of the incubation period compared to the FF treatment and the control group except 2% TTO level. In the same context, the results of 3% level of both oils are better than a 2% level, as well, LavO at both doses (2 and 3%) enhanced fetal weight significantly compared to TTO. Spraying quail eggs after collection with TTO or LavO oils showed a significant decreased in yolk sac remaining weight (*P* < 0.01) at 14th day of incubation compared to the FF or control group except the TTO 2% level which did not significantly difference than FF and this decrease increases with the high doses of oil, while FF treatment had no effect on the yolk sac weight compared to the control. Egg-shell weight and egg-shell thickness on 14th day of incubation period revealed that these two measurements decreased significantly (*P* < 0.01) as a result of treating fertilized eggs with either oils or FF compared to the control, with a preference for oils treatments, the results also showed that low and high doses of LavO had the highest effect on these two measurements than TTO doses.

**Table 3:** The effect of spraying fertile quail eggs with tea tree (TTO) and lavender oils (LavO) before incubation on embryonic development and egg weight loss measurements

Traits	Egg weight (g) (14th day)	Egg weight loss % (0–14 day)	Embryonic weight (%)	Remaining Yolk sac weight %	Shell weight (%)	Shell thickness with membrane (mm)
Treatment						
Control	10.54	11.57^a^	41.80^e^	39.93^a^	8.26^a^	0.25^a^
EA 70%	10.61	10.8^ab^	45.98^cde^	37.18^ab^	6.84^b^	0.22^bc^
FF	10.6	10.92^ab^	43.47^de^	39.56^a^	6.97^ab^	0.23^b^
TTO 2%	10.61	10.76^ab^	46.92^cd^	36.52^ab^	6.56^b^	0.21^c^
TTO 3%	10.63	10.59^b^	53.32^ab^	30.45^cd^	6.32^b^	0.19^de^
LavO 2%	10.62	10.75^ab^	49.95^bc^	33.68^bc^	6.37^b^	0.20^cd^
LavO 3%	10.64	10.36^b^	56.64^a^	27.68^d^	5.69^b^	0.18^e^
SEM	0.23	0.18	1.41	1.50	0.35	0.01
*P*-value	1.000	0.024	0.000	0.000	0.016	0.000

^a, b, c, d, e^means within each column for each item with different superscripts are significantly different (*P* ≤ 0.05). *SEM* Standard error of means, *P* Probability level

Results of fertility, hatchability and embryonic mortality rate are shown.

in Table (4). The percentage of fertilized eggs in all study groups were about 90%, meaning that there was no effect of spraying eggs with TTO and LavO oils or fumigating formaldehyde on fetal mortality before or at the beginning of incubation. Spraying fertilized eggs with TTO or LavO at levels of 2 and 3% before incubation significantly boosted (*P* < 0.01) the hatching rate recording 3.4, 8.34% for the TTO treatment and 6.68, 10.36% for LavO above the FF treatment rate, respectively, while these increases recorded 6.12, 10.65% for TTO and 8.96, 12.72% for LavO above the control treatment rate, respectively. On the other hand, fumigating fertilized eggs with formaldehyde increased the hatching rate compared to the control group, but this improvement was not significant. Data on embryo mortality rates during incubation periods (0–4 days, 5–15 days, 16–18 days) or from 0–18 days showed that spraying TTO or LavO on fertilized hatched eggs immediately upon collection led to a significant reduction (*P* < 0.01) in embryonic mortality rates during incubation compared to the FF treatment except the TTO 2% level during 5-15th day, while, FF treatment had a significant reducing effect (*P* < 0.01) on this measurement compared to the control group. As well, it could be noted that LavO had a higher effect on reducing the embryo mortality rate compared to TTO, and within a single oil, the embryonic mortality rate decreased significantly (*P* < 0.01) with an increase oil dose.

**Table 4:** Effect of spraying hatching quail eggs with tea tree and lavender oils before incubation on percentages of hatchability, fertility and embryonic mortality

Traits	Hatchability of fertile eggs	Fertility (%)	Embryonic mortality (%)			
Treatment			0–4 d	5–15 d	16–18 d	0–18 d
Control	84.63^f^	90.30	5.17^a^	7.17^a^	3.00^a^	15.37^a^
EA 70%	87.95^de^	90.97	4.67^ab^	5.67^b^	1.67^b^	12.05^c^
FF	86.44^ef^	90.67	4.33^b^	6.33^ab^	2.67^a^	13.56^b^
TTO 2%	89.81^cd^	90.97	3.00^c^	5.67^b^	1.67^b^	10.19^d^
TTO 3%	93.65^ab^	90.67	1.00^e^	4.00^c^	1.33^b^	6.34^f^
LavO 2%	92.22^bc^	90.30	2.00^d^	4.67^c^	1.00^b^	7.77^e^
LavO 3%	95.40 ^a^	90.97	1.00^e^	2.33^d^	1.00^b^	4.60^g^
SEM	0.83	87.58	2.51	0.26	0.19	0.52
*P*-value	0.000	0.990	0.000	0.000	0.000	0.000

^a, b, c, d, e^means within each column for each item with different superscripts are significantly different (*P* ≤ 0.05). *SEM* Standard error of means, *P* Probability level

In general, from Table 5, treatment of fertilized eggs with TTO and LavO (at 2% and 3% levels) immediately after collection improved chick quality measurements on the first day compared to the control group or the FF group, as chick body weight (g), chick length (cm), activity (%), down and appearance (%), closed navel (%), no residual membrane (%) and no residual yolk (%) were improved significantly (*P* < 0.01) in the TTO and LavO groups and this improvement was oil dose dependent manner (Table 5). In the same context, the 2% TTO level has a higher positive effect in improving chick measurements than the 2% LavO level, while at the 3% dosage the preference was for LavO. All experiments eggs were subjected to the same ideal temperature and humidity conditions, so the variable chicks' weight gain at hatching chicks were due to treatments.

**Table 5:** Effect of spraying hatching quail eggs with tea tree and lavender oils before incubation on chick quality traits

Traits	Chick weight at hatch (g)	Length (cm)	ActivityGood (%)	Downs and Appearance (%)	Closed navel (%)	No remaining membrane (%)	No remaining yolk (%)
Treatment							
Control	7.95^d^	9.10^f^	86.24^e^	89.86^c^	87.21^c^	86.17^b^	91.59^b^
EA 70%	8.34^d^	9.97^d^	91.34^cd^	92.34^bc^	91.90^ab^	97.03^a^	97.38^a^
FF	8.27^ed^	9.54^e^	89.43^de^	91.93^bc^	89.13^bc^	97.03^a^	97.47^a^
TTO 2%	8.50^cd^	10.36^cd^	95.21^ab^	96.79^a^	93.34^a^	98.59^a^	97.52^a^
TTO 3%	9.02^b^	10.91^b^	96.59^ab^	97.24^a^	95.28^a^	98.59^a^	97.55^a^
LavO oil 2%	8.72^bc^	10.50^bc^	93.83^bc^	95.07^ab^	92.21^ab^	97.24^a^	96.86^a^
LavO oil 3%	9.54^a^	11.37^a^	97.41^a^	97.24^a^	95.00^a^	98.59^a^	97.41^a^
SEM	0.11	0.14	1.07	1.06	1.14	0.76	0.88
*P*-value	0.000	0.000	0.000	0.000	0.000	0.000	0.000

^a, b, c, d, e, f^means within each column for each item with different superscripts are significantly different (*P* ≤ 0.05). *SEM* Standard error of means, *P* Probability level

Some organs relative weights (liver, heart, gizzard, intestine, gallbladder and remaining yolk sac of Japanese quails’ chicks at 1 day of age are presented in Table 6. There was a non-significant increase in the relative weight of the heart, gizzard, intestine, and gallbladder in the TTO and LavO groups compared to the control or FF group, and this numerical increase depended on the oil level, with a preference for the 3% level of LavO. On the other hand, there was a significant reduction in the liver and the yolk sac relative weight due to subject eggs to TTO, LavO oils or FF treatment compared to the control group, and this decrease was oil dose dependent.

**Table 6:** Effect of spraying hatching quail eggs with tea tree and lavender oils before incubation on some chick organs relative weights

Traits	Liver (%)	Yolk sac (%)	Heart (%)	Intestine (%)	Gallbladder (%)	Gizzard (%)
Treatment						
Control	3.35^a^	14.53^a^	0.71	3.67	0.15	5.47
EA 70%	2.39^b^	11.95^ab^	0.81	3.92	0.24	5.58
FF	2.37^b^	12.82^ab^	0.80	3.80	0.16	5.64
TTO 2%	2.51^b^	11.07^abc^	0.90	4.01	0.30	5.77
TTO 3%	2.62^b^	9.76^bc^	1.07	4.23	0.42	6.38
LavO oil 2%	2.57^b^	10.57^bc^	1.01	4.01	0.33	6.23
LavO oil 3%	2.75^b^	7.83^c^	1.13	4.73	0.43	6.74
SEM	0.18	0.99	0.10	0.24	0.08	0.33
*P*-value	0.035	0.024	0.079	0.185	0.194	0.132

^a, b, c^means within each column for each item with different superscripts are significantly different (*P* ≤ 0.05). *SEM* Standard error of means, *P* Probability level

**Fig. 1:**
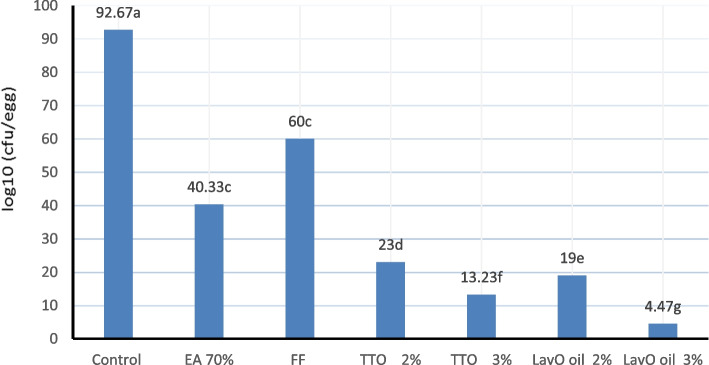
The effect of spraying fertile quail eggs with tea tree (TTO) and lavender oils (LavO) before incubation on bacteriological activity of eggshell surface. The values are represented by vertical bars. ^a,b,c,d,e,f,g^Mean values with unlike letters were significantly different (*p* < 0.01)

The results presented graphically in Figure (1) illustrated the total bacterial count on the surface of the fertilized eggshell (log^10^ (CFU g^−1^)). The results showed that spraying fertilized eggs immediately after collection with TTO or LavO at low and high doses affected bacterial growth on the eggshell surface, resulting in a significant reduction (*P* < 0.01) in the total bacterial count compared to the FF treatment, which also caused a significant decrease in the total bacterial count compared to the control group. As well, between the two essential oils the preference was for LavO.

## Discussion

To produce the highest quality day-old chicks, hatching is an important stage in the poultry industry as it represents a crucial step that improves the efficiency of the poultry sector. The interest in the poultry production field is no longer limited to increasing poultry production rates only, as increasing performance rates has become linked to preserving the environment and the health of poultry workers. Based on previous studies, the quality of fertilized eggs must be maintained not only immediately after laying, but also from the moment they develop in the hen’s oviduct. Whereas, obviously, disinfection results are greatly affected by the type of disinfectant as well as the timing of treatment [13]. According to Mayes and Takeballi, [42], dirty nests and cages can serve as sources of contamination to eggs. In addition to surface contamination, infectious organisms can be transmitted from an infected hen to the egg during fertilization, development of the egg in the hen's oviduct or immediately after the egg is laid upon contact with contaminated feces or bedding causing contaminated eggs became wet and warm. Cason et al*.,* [43] demonstrated that there are numerous infectious organisms that can infect an egg before and after laying and *Escherichia coli* is the most common isolated bacterium that could enter the egg from an infected hen' reproductive tract or penetrating the eggshell if the egg is contaminated with feces, which caused yolk sac infection, leading to a watery and yellow–brown or yellow-green content. As noted by Copur et al. [13], there is no effective method to eliminate microorganisms after they have crossed the membranes of eggs, or to prevent their further invasion of the egg contents or their deleterious effect on embryonic development. In the field application, fumigation, UV irradiation, spraying and washing with appropriate disinfectant are common applied practices for sanitation [44–46], so, disinfection of the eggshell surface prior to incubation is an essential control practice for obtaining high-quality hatching performance and thus preventing diseases in the farm [47]. To achieve this goal, fumigation of hatching eggs using paraformaldehyde is commercially used by most producers [6]. In spite of, this technique is efficient to reduce the harmful hazardous of the potential pathogenic microorganisms [7] but formaldehyde is toxic and has adverse effects not only for chick embryos but also for human health and environment (6 and 8) and they cannot tolerate constant exposure, especially at high concentrations, because of potential health risks [26–29]. As Zeweil et al*.,* [8] reported hatchery sterilization with paraformaldehyde causes deformities in the development of chick embryos.

Our result explained that neither LavO nor TOO led to changes in external traits (egg weight, eggshell weight, eggshell thickness and egg shape index) or internal traits (yolk and albumin weight, yolk index, Haugh unit and yolk color) egg quality traits during 5 consecutive storage days from egg laying to the start of the incubation period. The present result was obtained because the eggs were obtained from quail hens at the same age that received the same conditional administration at home.

Our data revealed that disinfect fertilized eggs with TTO and LavO oils had a considerable effect on decreasing the percentage of egg weight loss during the incubation period (from 0 to 14th day), which coincided with increasing the percentage of embryonic weight compared to the FF treatment or the control, and this effect was significant (*P* < 0.05) with the higher oil doses. In our study, since all experimental eggs were taken from the same flock, the results of eggshell weight and thickness did not show significant differences between TTO, LavO, FF and control groups before the eggs were introduced into the incubator, indicating that the lower egg weight loss in TTO and LavO groups after incubation was due to the closure of eggshell pores by the tested oils, which caused a decrease in water evaporation. Our concept was identical with Shahein and Sedeek, [4] who noticed that the low percentage of egg weight loss during the hatching period was due to the oily nature of propolis disinfectants, which works to close the pores of the eggs, which leads to reducing water evaporation. Thus, the variable results of egg weight loss percentage due to egg treatment with disinfectants are reasonable since the disinfectants might affect the cuticle layers and shell porosity. Additionally, the reducing egg weight loss percentage in our experiment groups might be attributed to the treatment eggshell with either TTO or LavO prevent cuticle layer destroyed and the egg pores coated with oil resulted in reducing the evaporation of water, whereas, excessive internal water loss can lead to severe dehydration in the fetus. Our results were in harmonization with the previous results that explained, too-fast moisture loss from incubating eggs were disadvantageous for the normal embryonic development [48] and this loss was approximately 12–14% of initial egg mass till pipping time, which an important factor influencing hatching success, in many domesticated species [49, 50]. Moreover, poor eggshell quality has been related to a higher percentage of egg moisture loss during incubation [51] and low hatchability [52]. As well, Oliveira et al., [2] reported that egg weight loss from fertilized eggs disinfected with clove essential oil was within the ideal range reported in the literature [53], resulting in heavy chicks in the Pasgar score assessment and had a superior effect on the physical quality of the chicks compared to undisinfected egg treatment due to the high rate of bacterial contamination that may occur, resulted in yolk sac infection [54]. in recent years, essential oils have been used in egg coatings to improve their structures and reduce weight loss in eggs stored for more than 20 days [55, 56]. On the other hand, the cuticle may be affected by the use of chemical disinfectants, which leads to changes in the development of the fetus [57].

Regarding to Embryo weight at 14 days of incubation (Table 3) was highly significant increased (*P* < 0.01) when spraying TTO and LavO at low or high dosage on fertilized eggshell surface directly upon collection compared to the untreated group or FF group. The increase embryo weight that occurred in TTO and LavO groups was attributed to TTO or LavO prevent cuticle layer destroyed and the egg pores coated with oil resulted in reducing the evaporation of water. As well as, the active anti-microbial substances present in TTO and LavO oils that prevent the penetrate microorganisms through the eggshell pores and their access to the internal egg contents, thus preventing their effect on the fetus growth. Whereas the increase in embryo weight in the oils treated groups coincided with a highly significant reduction in the total bacterial count on the surface of egg shell (Figure 1). The use of FF to disinfect fertilized eggs destroyed the microorganisms present on the surface of egg shell before eggs incubation, but did not prevent recontamination of the egg shell with microorganisms during the incubation period, while, disinfecting eggs with TTO and LavO oils destroyed the existing microorganisms on the egg shell surface, and this effect continues during the egg incubation period, in addition, the usage oils prevent microorganisms penetrating through the egg shell pores, which reduces the effect of microorganisms on the fetus growth, so, embryo weight was improved at 14th day of incubation. The previous literature explained that if hatching eggs are not disinfected prior to incubation, excessive bacterial contamination and subsequent growth of bacterial populations can result in reduced hatchability, poor chick quality, and poor growth and performance [58], for example, E. coli infection that has occurred during incubation development led to yolk sac infection (omphalitis) and poor weight gain. Essential oils have an effective effect in reducing microbial contamination of eggshells intended for incubation [13, 59], therefore, it is reasonable to expect such an effect by LavO due to its well-documented antimicrobial and antioxidant properties effects [21, 60]. As the same trend, Oliveira et al*.,* [2] mentioned that disinfecting fertilized eggs with clove essential oil reduces microbial populations and does not negatively affect the embryos. Additionally, heavier body weight of chicks at hatching was recorded for chicks produced from propolis-treated groups compared to those produced from the untreated group and the authors explained this result by the clogging of egg pores due to the oily nature of propolis disinfectants which resulted in lower egg weight loss during incubation [4]. According to Geng and Wang [48], during the incubation period, too rapid moisture loss through the eggshell pores was unfavorable for normal embryonic development.

The decrease of yolk sac remaining weight (Table 3) at the 14th day of the incubation of fertilized quail eggs treated with TTO or LavO at any studied doses referred to improve yolk absorption and utilization by the embryo, as the remaining yolk sac weight (%) was decrease significantly (*P* < 0.01) compared to the FF or control group, as well, this benefit was correlated to oil-dose dependent manner. While, FF treatment had no effect on the yolk sac remaining weight compared to the control, which did not differ between the two groups. Eggshell weight and eggshell thickness on 14th day of incubation period revealed that in general, subjecting fertilized quail eggs to TTO, LavO or FF increased the egg shell calcium absorption rate by embryos compared to the control, whereas, egg shell weight and egg shell thickness measurements decreased significantly (*P* < 0.01) as a result of treating fertilized eggs with either oils or FF, with a preference for oils treatments, the results also showed that LavO had the highest influence on these two measurements than TTO doses.

The lower embryonic mortality rate (Table 4) in the essential oils treated groups compared to the FF and control groups also demonstrates the improved hatching rate. Also, the increase in egg weight loss during incubation from (0–14 days) for the FF and control groups led to the worst hatching percentage. In contrast, the lower egg weight loss percentage for TTO and LavO was one of the reasons that explained the increase in the hatching percentage. Our results were in agreement with the finding of Fouad et al*.,* [19] who found that an incredible improvement in hatchability of total or fertilized eggs was observed when hatching eggs were sprayed with garlic oil during the first 10 days of incubation compared to untreated or water-sprayed eggs. From the previous literature, eggs with the lower weight loss had higher hatchability and eggs with greatest weight loss had the lowest hatchability [61], this conception was supporting our results that in a harmonization with the finding of Shahein and Sedeek, [4] who mentioned that the highest hatchability percentage of fertilized eggs disinfected with propolis was correlated with the decrease egg weight loss during 1–18 day of incubation and the opposite was true in the control group. As well, better hatchability may be a direct result of reduced microbial contamination of eggs treated with TTO and LavO. These results were in agreement with other studies that used alternative natural products to disinfect eggs such as allicin [17], propolis [4], garlic extract [18] and garlic oil [4, 19], which led to improved hatching rates. If hatching eggs are not disinfected before incubation, excessive bacterial contamination and Subsequent growth of bacterial populations can result in reduced hatchability, poor chick quality, and poor growth and performance [58]. Failure to disinfect hatching eggs prior to incubation causes excessive bacterial contamination and subsequent growth of bacteria, resulting in reduced hatchability, poor growth, and poor chick quality and performance [58]. At the same context, Yildirim et al*.,* [37] who reported that a significant difference between oregano vulgaris and formaldehyde fumigation in the hatchability of fertile eggs was recorded. According to the results of Copur et al*.,* [13]; Ulucay and Yildirim, [59] the essential oils were effective in reducing microbial contamination of incubating egg shells, which is consistent with the present results. The decrease of embryonic mortality rates during incubation periods (0–4 days, 5–15 days, 16–18 days) or from 0–18 in the two essential oils groups could be explained by the antimicrobial substance present in the two studied oils destroyed microorganisms that found on eggshell surface and prevent penetrate them into egg content that effect on growth of embryos or kill embryos through incubation period. From the present data we could also noticed that the effect of oils antimicrobial substances on destroying microorganisms began from the moment the eggs were treated immediately after laid and during the period of keeping the eggs before incubation and during the incubation period, while treatment with formaldehyde effect began immediately before the eggs were incubated and its effect ends after a period of incubation of the eggs, and regrowth of microbes on the eggshell can occur if the incubator is contaminated and not well disinfected. The present results were in a good agreement with the results of Fouad et al*.,* [19]; Abo-Samaha and Basha [1], they explained that garlic oil enhances embryo growth and significantly reduces early, intermediate and late embryo mortality when quail eggs treated with garlic oil, because garlic oil contains an effective antibiotic, anti-inflammatory and antioxidant component. As like, disinfecting hatching eggs using of allicin as an alternative disinfectant lowered the early and late embryonic mortalities compared to FF or non-treated group [17]. Shahein and Sedeek [4] reported that propolis and FF reduced embryonic mortality in the treated groups, and that increased embryonic mortality in the untreated control group is an indication of increased bacterial load on the eggshell surface and bacterial multiplication either on the shell surface or inside the eggs. The best significant results of TBC were observed for propolis, which had a residual effect on eggshell surface for longer time of storage, while FF did not possess the same character. In this context, Oliveira et al*.,* [2] the significant reduction in embryo mortality during the late incubation stage in eggs sprayed with clove essential oil compared to eggs sprayed with grain alcohol and the control group may be related to the reduction in the number of microbes on the eggshell due to the action of the chemical components present in the oil. The results of Gabriel et al. and Bakhit [62, 63] showed that sterilizing hatching eggs with FA increased the early embryonic mortality rate compared to essential oils, as this reduced the mortality rate during this period by about 75%, as the embryo benefited from the antibacterial barrier formed by essential oils on the eggshell.

The present results revealed that chick weights at hatching time and chick quality measurements (Table 5) were higher in the TTO and LavO compared to FF or control groups, on the other hand, the result of FF did not differ than the control group. These improvements could be attributed to TTO and LavO prevented the embryo dehydration resulting from the excessive weight loss of the eggs during incubation. As well, the fact that the essential oils are known to have antimicrobial properties, affecting the growth of microorganisms, destroying them and preventing their penetration into the entire egg components. Furthermore, when treating hatching eggs with two tested oils (TTO and LavO), perhaps they may become absorbed into the egg components, and since essential oils contain antioxidants, these compounds will be absorbed by the fetus, and their effect will be reflected on the enhance the embryo development with reducing the embryonic mortalities resulted in improve embryo growth, the chick' weight upon hatching, health status and the vitality of the chick. Therefore, essential oils have antibiotic, antioxidant and anti-inflammatory effects, which are attributed in particular due to the presence of phenolic and polyphenolic substances [21, 22, 64]. Our results are consistent with those obtained from the previous researches. Clove oil had a superior effect on the physical quality of chicks compared to the other treatments conducted to heavy chicks in the Pasgar score assessment [22, 53]. Lynn [65] mentioned that small chicks have a higher surface area to weight ratio thus they dry more easily than longer chicks. It is known that if the eggs designated for incubation are not disinfected with an effective disinfectant before being placed in the incubators, the quality of the hatched chicks may decrease due to the high bacterial contamination that may occur, which may cause inflammation of the yolk sac [54]. Therefore, the level of microbial contamination may be an indicator of chick quality.

The present results showed that spraying TTO and LavO essential oils on eggs immediately after collection had a highly significant disinfectant effect (Figure 1), while the bacterial count on treated eggshell was highly significant decreased and this decrease may be due to that these two essential oils destroyed the microorganisms present on the surface of the eggshell. In addition, the pores of the eggshell were closed with treated oils, which prevented the penetration of microorganisms through the pores of the eggshell into the internal contents of the egg during both the period of keeping the eggs in storage room, and this effect continued during the period of incubation of the eggs, beside decreased the water evaporation resulting in reducing the egg weight loss percentage and mortality percentage that reflected on increasing hatchability percentage and chick weight on hatching. Our finding was in accordance with that of Harry and Gordon [66], to maximize the efficiency of egg incubation operations and increase the quality of day-old chicks, microbial contamination must be minimized during egg incubation to help produce cleaner, healthier chicks. Smeltzer et al*.,* [67] reported that the eggshell contains many pores with diameters ranging from 9 to 35 μm, so pathogenic bacteria present on the surface of the egg may contaminate the eggshell and penetrate the egg through the pores of the shell [68]. According to Cook et al*.*, [69], when microorganisms penetrate the membranes of hatching eggs, there is no effective way to eliminate them or prevent their further invasion of the egg contents or embryo development. Therefore, harmful microorganisms must be removed or destroyed as quickly as possible on the surface of the hatching egg. If hatching eggs are not disinfected prior to incubation, excessive bacterial contamination and subsequent growth of bacterial populations can result in reduced hatchability, poor chick quality, and performance [58]. For example, E. coli infection that occurred during incubation resulted in yolk sac infection (omphalitis) and poor weight gain. It could be explained the antimicrobial effect of essential oil as follows, Essential oils have antimicrobial effect led to reduce eggshell contamination [13, 59] and this effect due to Phenolic compounds in essential oils, which interact with phospholipids present in the bacterial cell membrane, making it more permeable leading to loss of ions, decreased membrane potential, exhaustion of proton pump function, and decreased adenosine triphosphate, causing cell death [70]. It is reasonable to expect such an effect by LavO because of its well-documented antimicrobial and antioxidant effects [21, 60]. At the same way, Tea tree essential oil shows broad antimicrobial activity, which can be mainly attributed to its terpinen-4-ol content [71], which stimulates the leakage of cellular potassium ions and inhibits respiration in E. coli cell, causing cytoplasmic membrane damage [36]. According to Gabriel et al., [63], essential oils extracted from Ocimum basilicum, Citrus aurantifolia or Allium sativum were significantly effective in reducing eggshell contamination and showed superior control over recontamination, maintaining stable bacterial loads up to 18th day of incubation but FA were less effective in preventing recontamination. Likewise, Copur et al*.,* [13] found that harmful microorganisms must be removed or destroyed as quickly as possible on the surface of the hatching egg to avoid contamination of the eggshell and penetration through the pores of the shell [68]. Therefore, Oliveira et al*.,* [2] mentioned that clove essential oil prevents the presence of salmonella, and pathogenic E. coli in the eggshells after sanitation. disinfecting Japanese quail eggs with Moringa oil before incubation was good practice to reduce bacterial contamination on eggshell surface and improve embryonic development and hatchability [72].

## Conclusions and recommendations

The present study concludes that spraying LavO or TTO as a natural disinfectant for fertilized Japanese quail eggs immediately after collection from chicken nests instead of fumigation with formaldehyde can be a positive solution and a good practice to reduce egg weight loss and embryonic mortality, improve hatching rate as well as chick weight at hatching and chick performance with a preference of 3% level. They also provide a good alternative treatment for controlling the microbial load on the eggshell surface during incubation, as well as reducing the hazardous effects of formaldehyde on hatchery workers. The use of these two essential oils as a natural disinfectant for fertilized eggs needs further studies, while the 3% level gives the highest results, so the level can be increased above that used in the current study.

## Data Availability

No datasets were generated or analysed during the current study.
